# Use of ultrasonography for evaluation of stability of lateral compression type 1 (LC-1) pelvic fractures to assist determination of treatment strategy

**DOI:** 10.1186/s13018-018-1047-z

**Published:** 2019-01-07

**Authors:** Bin-Fei Zhang, Jin-Lai Lei, Hong Zhang, Peng-Fei Wang, Hu Wang, Yu-Xuan Cong, Hai Huang, Yan Zhuang

**Affiliations:** 1 Department of Orthopedic Trauma, Honghui Hospital, Xi’an Jiaotong University, 555 Youyi East Road, Beilin District, Xi’an, Shaanxi China; 2 Department of Ultrasound Medicine, Honghui Hospital, Xi’an Jiaotong University, 555 Youyi East Road, Beilin District, Xi’an, Shaanxi China

**Keywords:** Ultrasonography, Stability, LC-1 pelvic fractures

## Abstract

**Background:**

Lateral compression type1 (LC-1) pelvic fractures represent a wide spectrum of heterogeneous injuries. These include both stable and unstable patterns; however, determining whether a LC-1 fracture is stable or unstable is a challenge, and the method used to evaluate fracture stability is complicated.

**Methods:**

We prospectively collected and analyzed data from 22 patients with LC-1 pelvic fractures, who underwent ultrasonography and a pelvic compression and separation test, in order to evaluate the role of ultrasonography in determining fracture stability and assist decision-making for treatment strategy.

**Results:**

Twenty-two patients (15 men and 7 women) were included in the study. Following an ultrasound examination, 10 patients were classified into the stable group and 12 into the unstable group. In total, 13 patients received conservative treatment and 9 underwent surgery. At follow-up, there were no differences in fracture healing times or fracture-related complications between the two groups. The Majeed score was comparable between the two groups and most patients recovered well. There was a moderate degree of consistency in Kappa values (Kappa = 0.571, *P* = 0.01) between the classification of stability and the final treatment received. In addition, the sensitivity of ultrasonography was 66.67% and the specificity was 76.92%.

**Conclusions:**

In conclusion, ultrasonography is a useful tool for diagnosing the stability of LC-1 pelvic fractures and assists the determination of treatment strategy. Left-right mobility ≥ 0.3 cm may be used as the criterion for determining instability.

**Trial registration:**

ChiCTR-DDD-16008722.

## Background

Lateral compression type-1 (LC-1) pelvic fractures are the most common type of pelvic fractures, accounting for approximately 50% of all pelvic ring fractures [1]. An increase in the numbers of vehicles on the road is responsible for the increase in the occurrence of pelvic fractures [2]. Traditionally, LC-1 pelvic fractures have been defined as rotationally unstable and vertically stable. Most of these types of fractures could be conservatively treated to achieve a good functional outcome [3–5].

In reality, LC-1 fractures represent a spectrum of heterogeneous injuries with patterns ranging from stable to unstable [6]. In addition, Beckmann et al. found that there are currently vast differences in decision-making between surgeons when treating LC-1 injuries. Thus, determining whether the LC-1 fracture is stable or unstable is a challenge and deciding whether the injury requires surgery or not is complex. In the case of a common LC-1 fracture, we could not judge pelvic stability using only the information from static radiographs. Additionally, the method used to evaluate stability is complicated. Sagi et al. examined the stability of pelvic fractures under anesthesia [7] while Tosounidis et al. performed stress examinations of the pelvic ring using fluoroscopy under general anesthesia [8].

In our previous study, we used a combination of ultrasonography with the pelvic compression and separation test to assess stability in seven patients [9]. Four unstable patients were treated with anterior and/or posterior stabilization, and three stable patients were treated non-surgically. All patients had recovered well at time of the final follow-up.

Conservative treatment is not suitable for every patient with a LC-1 fracture. However, non-surgical treatment must be avoided for unstable pelvic fractures. If an unstable LC-1 pelvic fracture cannot be treated using suitable surgical fixation, the fracture may undergo late displacement or non-union. Bruce et al. [10] reports that LC-1 fractures that were not operated on displaced at a rate of 8.4% in the follow-up period. Van den Bosch et al. [11] reported that three LC-1 fractures developed non-unions and that non-union appears to occur most often following conservative or suboptimal surgery of unstable pelvic ring fractures. Compared with early fixation, late reconstruction is more complex and technically much more demanding [12]. Thus, it is very important to distinguish stable and unstable LC-1 pelvic fractures during the acute stage of pelvic ring injuries.

Using ultrasound examination, it is relatively easy to distinguish between a stable and an unstable pelvic fracture. When a LC-1 pelvic fracture is shown to be stable, it enhances our confidence in choosing conservative treatment; if the fracture is shown to be unstable, it reminds us that this patient is at risk of developing a late displacement or non-union. We should focus on these patients at follow-up and consider the importance of further consultation. In the case of obviously unstable fractures, we suggest early surgery to prevent poor outcomes later.

In this study, we analyzed the data from LC-1 pelvic fracture patients, combining ultrasonography with the pelvic compression and separation test to evaluate the role of ultrasonography in distinguishing stability and assisting in determination of treatment strategy.

## Methods

### Ethical statement

The study was approved by the Ethics Committee of the Xi’an Jiaotong University (No. 2016053). Each patient provided informed consent before ultrasonographic examination. In addition, this study was performed in concordance with the international ethical guidelines for studies involving human subjects, according to the Declaration of Helsinki [13].

### Patients

Inclusion criteria for the study required that patients met the diagnostic criteria for LC-1 pelvic fractures [14]. Patients must have had a history of falling, stumbling, or a traffic accident followed by pelvic pain, tenderness, dysfunction, and local swelling. Diagnosis and fracture type were confirmed using X-ray and computed tomography (CT). X-ray and CT images revealed partially stable fractures with a lateral-compression injury in the pelvis. Patients with records of ultrasonography and pelvic compression and separation tests and at least 6 months of follow-up were included in the study.

### Study protocol

**Fig. 1:**
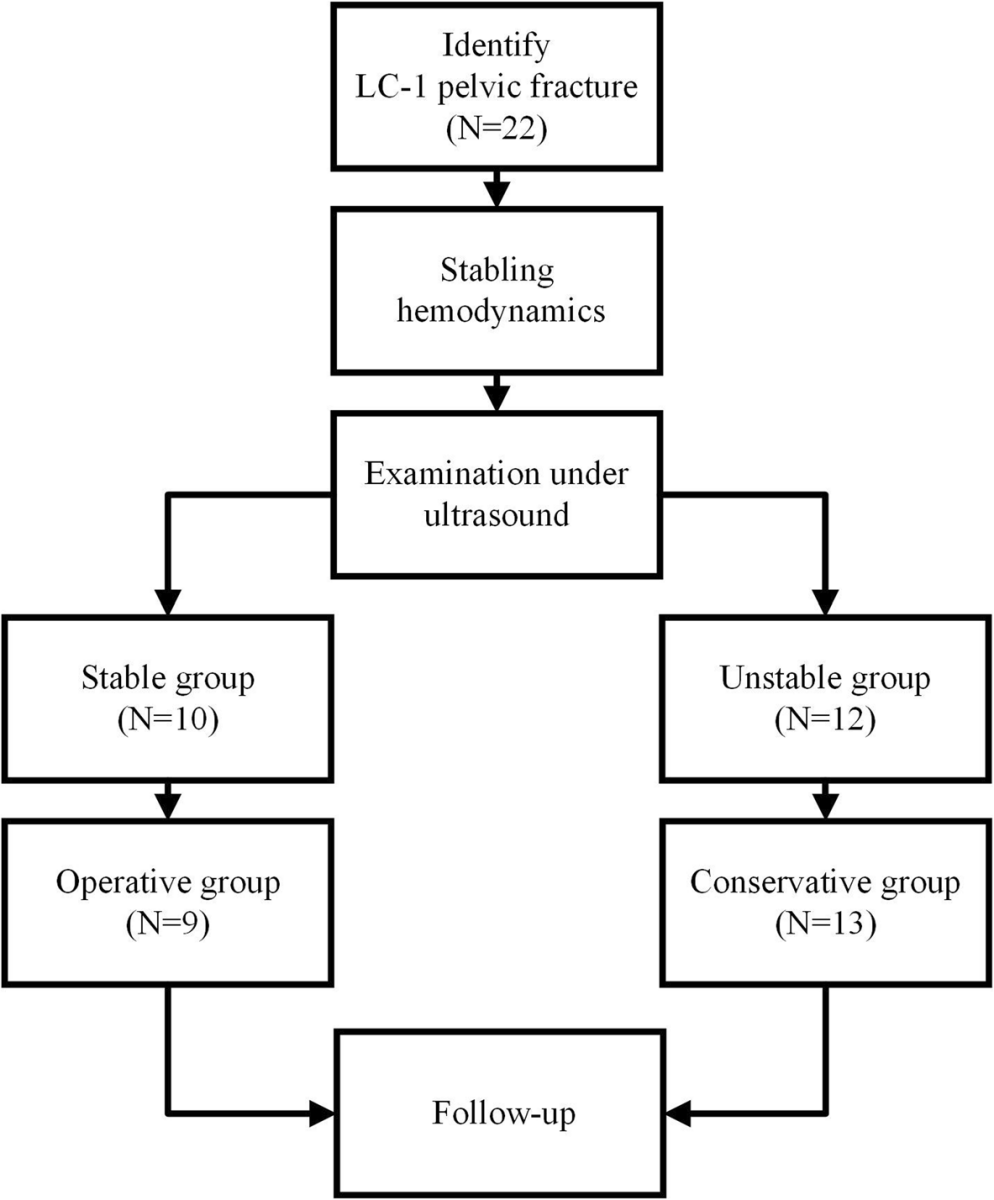
Flowchart showing study design and assignment of patients into groups

The flowchart of the study design and assignment into groups is shown in Fig. 1. When a LC-1 pelvic fracture was identified, we first ensured that the patient’s hemodynamics were stable, as in our previous study [9]. Next, pelvic stability was tested using the pelvic compression and separation test on the injured superior pubic ramus. The method and protocol were performed according to our previously published method [9]. Patients were tested by a senior ultrasound sinologist (Hong Zhang) and orthopedist (Bin-Fei Zhang). Video material was collected from the ultrasound system in order to compare the relative positions of the fracture sites in patients during rest, under compression, and during separation to determine fracture stability. The detailed formula for calculating mobility is described in the methods section of our previous publication [9].

**Fig. 2:**
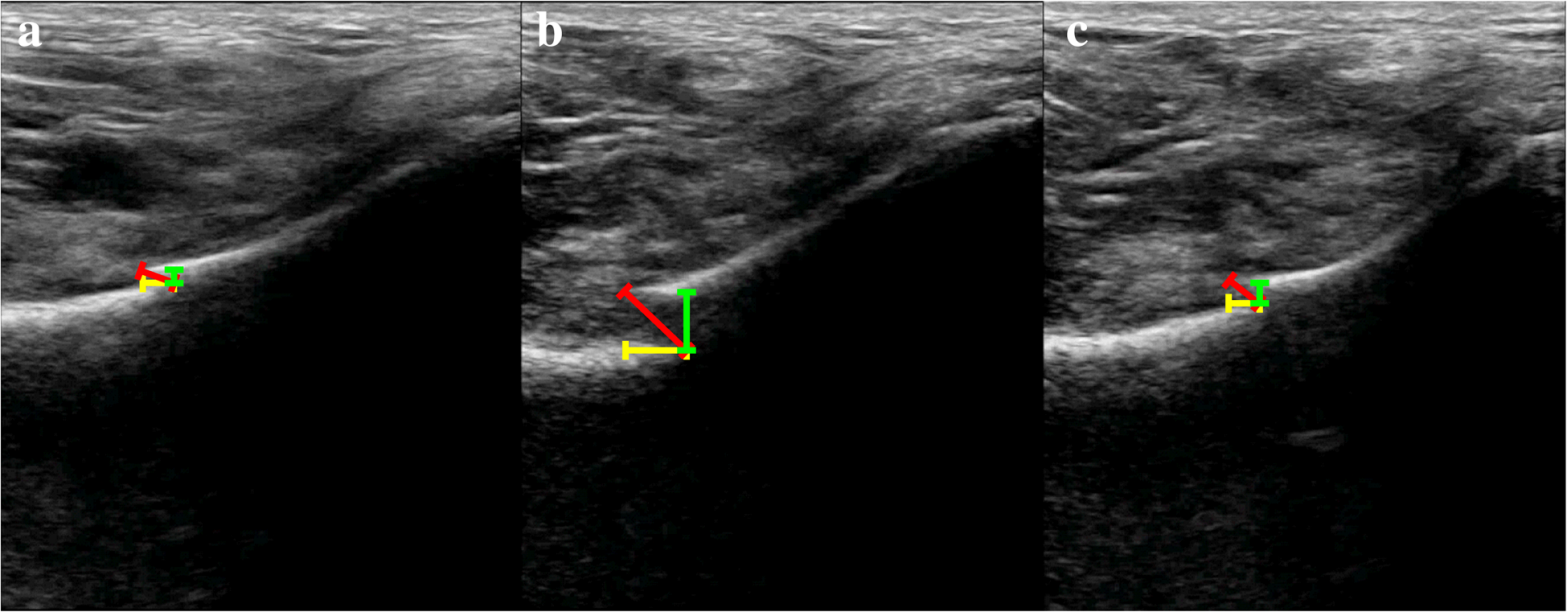
Method of measuring displacement in three directions, performed measures the displacement under the ultrasonic probe while the orthopedist performs the pelvic compression and separation tests. **a** Displacement at rest. **b** Displacement during the compression test. **c** Displacement during the separation test. The yellow line shows the L-R distance, the green line shows the A-P distance and the red line shows the oblique distance

In addition, we calculated the mobility of the fracture in three directions, including left-right (L-R mobility), anterior-posterior (A-P mobility), and oblique (oblique mobility). Figure 2 illustrates the method used to measure displacement in three directions.

Following the ultrasonography examination, we calculated the mobility and recorded the degree of patients’ pain using the visual analog scale (VAS). We divided the patients into two groups, a stable group and an unstable group, using L-R mobility ≥ 0.3 cm as the definition of instability based on our previous experience [9] and Tile’s criteria [15].

Treatment strategies were formulated by five senior surgeons, based on the mechanism of injury, fracture classification, pain, displacement on X-ray or CT images, and patients’ demand, among other factors, as well as results of ultrasound imaging.

When the final treatment was determined, patients were divided into an operative group and a conservative treatment group. In those patients who required surgery, the ilioinguinal approach or the Stoppa approach or close fixation was selected [16, 17], taking into account individual differences and injury type. During surgery, the camera captured images of the relative movement of fractures at rest and during the compression and separation test. In the conservative group, following 3–4 days of provision of pain relief, the patients were either sent home or to a community clinic, until the patient was tolerant of mobilization or weight-bearing on the affected side.

The follow-up frequency for these patients was at least once a month. All patients’ functions were evaluated using the Majeed grading system [18]. In addition, X-ray images were used to observe healing or new displacement. The time to weight-bearing was determined according to the degree of healing achieved.

The gold standard for assessing ultrasonography was healing of fractures in both the stable and the unstable groups.

### Statistical analysis

Statistical analysis was performed using SPSS (Version 19.0, SPSS Inc., Chicago, IL, USA). First, we assessed whether the measurement data was normally distributed using the Shapiro–Wilk test. We then analyzed the data using independent sample *t* tests or the Mann-Whitney *U* test. The enumeration data was processed using the chi-square test or Kappa test for consistency. Differences were considered to be statistically significant if a *P* < 0.05 was obtained.

## Results

### Patient characteristics

In total, 22 patients with LC-1 pelvic fractures were included in the study, between July 1, 2016 and March 31, 2017. The average age of patients (15 men and 7 women) was 53.05 ± 16.89 years (range 20–84 years). Electrocardiography monitoring was performed when the patients were admitted to the hospital, and we ensured that blood pressure and heart rates were stable.

### Assessment of pelvic stability by ultrasonography

Once the patient’s hemodynamics were stable and the routine X-ray and CT images had been performed to assess the fracture type, pelvic stability was tested using an ultrasound examination. The L-R mobility was subsequently calculated. According to the criterion of L-R mobility ≥ 0.3 cm, we divided the patients into two groups, the stable group and the unstable group. Detailed patient information is shown in Table 1. There were no differences in gender, age, mechanism of injury, comprehensive classification, or medical morbidity between the two groups. All the patients suffered mild to moderate pain at the pubic branch or sacroiliac joint during the pelvic compression and separation examination.

**Table 1:** Patient characteristics according to stability group

	Stable group	Unstable group	Total	*P*
No. of patients	10	12	22	
Gender				
Male	6	9	15	0.287
Female	4	3	7	
Age (years)	52.40 ± 18.83	53.58 ± 16.33	53.05 ± 16.89	0.921
Mechanism of injury				
Accident	5	3	8	0.097
Injury falling from a height	2	6	8	
Stumbling	3	3	6	
Comprehensive classification				
Unilateral pubic branch fracture	4	1	5	0.083
Bilateral pubic branch fracture	0	0	0	
Incomplete sacral + unilateral pubic branch fracture	3	3	6	
Incomplete sacral + bilateral pubic branch fracture	1	2	3	
Complete sacral + unilateral pubic branch fracture	1	4	5	
Complete sacral + bilateral pubic branch fracture	1	2	3	
VAS	2.70 ± 0.92	3.58 ± 1.35	3.18 ± 1.24	0.105
Medical morbidity				
Hypertension (%)	2	1	3	0.571
Diabetes (%)	0	1	1	1.000
Stroke (%)	1	0	1	0.455
Multiple injuries (%)	6 (60)	9 (75)	15	0.791
Deep vein thrombosis (%)	6 (60)	7 (58)	13	0.973

The displacement distances of the fractures were measured during rest, compression, and separation and mobility in three directions was calculated. Results are shown in Table 2. The distances between the fracture fragments in the unstable group were greater than those in the stable group in all directions (L-R, A-P, oblique) during both rest and compression. However, no differences in separation were observed between the two groups. It is important to note that the mobility of the unstable group in the L-R, A-P, and oblique directions were greater than in the stable group.

**Table 2:** Distance and mobility in three directions in stable and unstable group from the ultrasound

	Stable group (*n* = 10)	Unstable group (*n* = 12)	*P*
L-R distance during rest (cm)	0.22 ± 0.08	0.41 ± 0.28	0.038
A-P distance during rest (cm)	0.16 ± 0.06	0.37 ± 0.23	0.050
Oblique distance during rest (cm)	0.26 ± 0.10	0.58 ± 0.38	0.015
L-R distance under compression (cm)	0.25 ± 0.12	0.58 ± 0.27	0.001
A-P distance under compression (cm)	0.16 ± 0.07	0.41 ± 0.21	0.003
Oblique distance under compression (cm)	0.29 ± 0.13	0.71 ± 0.29	0.001
L-R distance under separation (cm)	0.19 ± 0.08	0.36 ± 0.38	0.356
A-P distance under separation (cm)	0.15 ± 0.08	0.29 ± 0.21	0.248
Oblique distance under separation (cm)	0.26 ± 0.10	0.52 ± 0.45	0.166
L-R mobility (cm)	0.11 ± 0.05	0.39 ± 0.22	0.001
A-P mobility (cm)	0.07 ± 0.06	0.21 ± 0.14	0.012
Oblique mobility (cm)	0.12 ± 0.07	0.40 ± 0.28	0.006

### Treatment for patients included in the study

In total, 13 patients received conservative treatment, and 9 received surgery. There was one patient in the stable group who underwent surgery, and four patients in the unstable group who did not undergo surgery.

There were no differences in sex, age, mechanism of injury, comprehensive classification, or medical morbidity between patients who underwent surgery or those who received conservative treatment, as shown in Table 3. In the operative group (4.00 ± 1.41), the VAS was higher than in the conservative group (2.62 ± 0.77; *P* = 0.015). A-P and oblique mobility in the operative group was greater than in the conservative group; however, there was no difference in L-R mobility. In total, nine patients underwent surgery, including six patients who received plates and three who received channel screws. Five patients received only one plate in the anterior ring using the Stoppa approach. One patient received one plate anteriorly using the ilioinguinal approach and pedicle screws posteriorly using the posterior midline approach. Two patients received a unilateral superior pubic ramus cannulated screw and a unilateral sacroiliac screw. One patient only received an anterior ring cannulated screw. Intraoperatively, the movement of the fragments was examined using direct vision or C-arm X-ray. Furthermore, the patients were examined using X-ray to ensure that the fracture was fixed, and the location of plates or screws was appropriate. The average operative time was 102.78 min (range 50–210) when treating pelvic fractures. Intraoperatively, two patients received two units of packed red blood cells each.

**Table 3:** Patient characteristics according to final treatment

	Conservative group	Operative group	*P*
No. of patients	13	9	
Gender			
Male	8	7	0.735
Female	5	2	
Age (years)	55.38 ± 20.89	49.67 ± 9.50	0.385
Mechanism of injury			
Accident	5	3	0.219
High falling injury	3	5	
Stumble	5	1	
Comprehensive classification			
Unilateral pubic branch fracture	5	0	0.143
Bilateral pubic branch fracture	0	0	
Incomplete sacral + unilateral pubic branch fracture	3	3	
Incomplete sacral + bilateral pubic branch fracture	2	1	
Complete sacral + unilateral pubic branch fracture	1	4	
Complete sacral + bilateral pubic branch fracture	2	1	
L-R mobility (cm)	0.20 ± 0.13	0.36 ± 0.29	0.181
A-P mobility (cm)	0.08 ± 0.08	0.23 ± 0.13	0.007
Oblique mobility (cm)	0.16 ± 0.16	0.42 ± 0.28	0.008
VAS	2.62 ± 0.77	4.00 ± 1.41	0.015
Weight-bearing after the final treatment (month)	1.00 ± 0.58	1.55 ± 1.67	0.415
Time fracture healing (month)	3.00 ± 0.81	3.11 ± 0.78	0.606
Complication-related fracture	0	1	0.409
Majeed score (last follow-up)	81.62 ± 11.76	80.22 ± 10.51	0.687

The average follow-up ranged from 8 to 15 months, with a range of 11.07 ± 1.93 months in the conservative group and 11.78 ± 3.03 months in the operative group; the difference was not statistically significant (*P* = 0.441). The time to beginning of weight-bearing following the final treatment was 1.00 ± 0.58 months in the conservative group and 1.55 ± 1.67 months in the operative group, respectively. Fracture healing was observed in all patients. There was no difference in fracture healing time or rate of complications related to the fracture between the two groups. In the operative group, one patient suffered delayed healing. None of the patients required reoperation or revision. The Majeed score at the last follow-up was comparable in both groups, and most patients recovered well, as shown in Table 3.

### Evaluation of the ultrasonography diagnosis

**Fig. 3:**
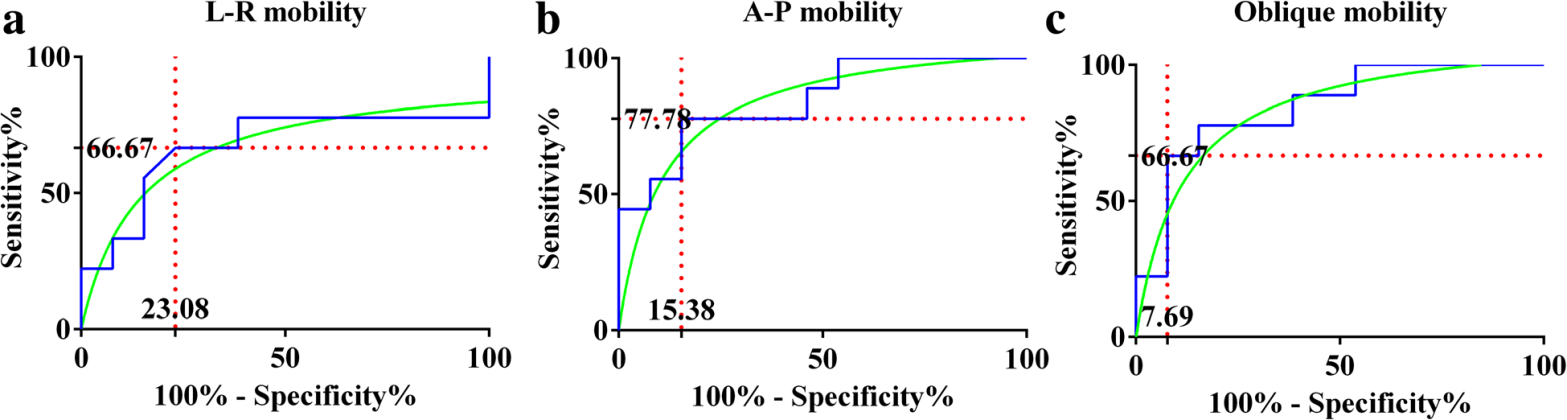
ROC curve for L-R mobility (**a**), A-P mobility (**b**), and oblique mobility (**c**), respectively

The Kappa test revealed moderate consistency (Kappa = 0.571, *P* = 0.01) between the result of the stability assessment and the final treatment, shown in Table 4. We drew the receiver operating characteristic (ROC) curve for the ultrasonography diagnosis. The cut-off value was 0.315 cm (area under the ROC 0.671) in L-R mobility, 0.140 cm (area under the ROC 0.850) in A-P mobility, and 0.270 cm (area under the ROC 0.842) in oblique mobility, as shown in Fig. 3. According to the cut-off value for L-R mobility, the sensitivity was 66.67% and the specificity was 76.92%.

**Table 4:** Consistency between pelvic stability and treatment strategy

Pelvic stability under ultrasonography	Final treatment		Total
	Operative	Conservative	
Stable	1	9	10
Unstable	8	4	12
Total	9	13	22

## Discussion

Sagi et al. found that 65% of LC-1 fractures were stable and 35% were unstable under general anesthesia [7]. Tosounidis et al. defines an overlap of the pubic rami fragments of 2 cm, or a similar overlap in the symphysis pubis under stress examination to be an unstable pelvis [8]. Shlamovitz et al. suggests that conscious patients who do not experience pelvic pain or tenderness are likely to have a stable pelvic fracture [19]. In addition, Olson et al. defined a stable pelvis as one that was able to “withstand the physiologic forces incurred with protected weight bearing, and/or bed to chair mobilization without abnormal deformation of the pelvis, until bony union or soft tissue healing could occur” [20]. There is a need for a good definition of pelvic stability, particularly during dynamic examination [6].

There is still considerable variability between trauma surgeons regarding the optimal or “required” treatment for many of the incomplete injury patterns [6], including the LC-1 type. Initial static X-ray and CT scan images only record a moment, and are unable to show the total amount of dynamic displacement that may have occurred during a traumatic event [7]. For these reasons, it is reasonable to surmise that pelvic stability will not be displayed on all pelvic radiographs. In this study, we report on a series of patients with LC-1 pelvic ring injuries for whom pelvic examination was performed using ultrasonography during the compression and separation test, in order to better characterize the pelvic stability in ultrasonography and determine instability of the pelvis for surgery.

In the unstable group, four patients received conservative treatment. The reasons for this were as follows: two patients requested non-operative treatment and two patients suffered femoral shaft or lumbar fracture requiring surgery, and therefore, we chose conservative treatment for the pelvic fracture. In the follow-up, four fractures healed and the Majeed score varied from 48 to 72. In the stable group, there was one patient who underwent surgery, due to persistent pain. In the follow-up, the patient’s Majeed score was 84.

Using ultrasound imaging, we can observe the fracture morphology, which is related to pelvic stability. The results have shown that more displacement is measured at rest and under compression, but not during separation. In the resting state, the injured hemi-pelvis remains in-balance with the main ligaments contributing to intact pelvic stability [21]. However, increased displacement leads to a greater possibility of instability, and we now understand how the fragments move under compression or separation. Following ultrasound examination, the movement and stability of the fracture can be observed and categorized. Specifically, in LC-1 fractures, the pelvis is internally rotated. Most fractures show the same deformity on the injured anterior ring. The fragments on the superior pubic ramus overlap. When the pelvis is compressed, the overlapping displacement will become increasingly obvious. When the pelvis is undergoing separation, the overlapping section in the stable group is more difficult to externally rotate, similar to the process of reduction in surgery.

We elected to calculate mobility in three directions using our formula [9], because L-R, A-P, and oblique commonly reflect displacement during compression and separation. Additionally, mobility in the L-R direction is the value that changes the most obviously.

According to Tile’s criteria [15], 0.3 cm was the cut-off value for judging the failure of fixation, and we used the L-R mobility ≥ 0.3 cm as the definition of instability at the beginning of the study. The final cut-off value calculated was 0.315 cm in L-R mobility, which should be used in future as a criterion to diagnose instability. As for the effectiveness of ultrasonography as a tool to assess instability of pelvic fractures, the result from the Kappa test showed a moderate degree of consistency (Kappa = 0.571). In additional, the sensitivity of ultrasonography was 66.67% and the specificity was 76.92%.

It is worth mentioning the limitations in this study. Firstly, this method could cause potential harm and pain to the patients; secondly, the muscle tension [9] and soft tissue conditions (obese vs. slim patients) are potential confounders to the results; thirdly, the correlation between stability and final treatment is weak; fourthly, different physicians for testing have different level of force, which is the other weakness of this study.

## Conclusions

In conclusion, ultrasonography is useful for diagnosing the stability of LC-1 pelvic fractures and assisting in the determination of treatment strategy. L-R mobility ≥ 0.3 cm may be used as the criterion of instability, but the sensitivity and specificity of this criterion was not high. The method must be confirmed in a large-scale controlled study.

## Abbreviations

A-P: Anterior-posterior
CT: Computed tomography
LC: Lateral compression
L-R: Left-right
ROC: Receiver operating characteristic
VAS: Visual analogue scale

## References

[1] Weaver MJ, Bruinsma W, Toney E, Dafford E, Vrahas MS (2012). What are the patterns of injury and displacement seen in lateral compression pelvic fractures?[J]. Clin Orthop Relat Res.

[2] Yoshihara H, Yoneoka D (2014). Demographic epidemiology of unstable pelvic fracture in the United States from 2000 to 2009: trends and in-hospital mortality[J]. J Trauma Acute Care Surg.

[3] Flint L, Cryer HG (2010). Pelvic fracture: the last 50 years[J]. J Trauma.

[4] Gordon RO, Mears DC (1991). Lateral compression injury of the pelvis. A case report[J]. J Bone Joint Surg (Am Vol).

[5] Gaski GE, Manson TT, Castillo RC, Slobogean GP, O'Toole RV (2014). Nonoperative treatment of intermediate severity lateral compression type 1 pelvic ring injuries with minimally displaced complete sacral fracture[J]. J Orthop Trauma.

[6] Beckmann JT, Presson AP, Curtis SH, Haller JM, Stuart AR, Higgins TF, Kubiak EN (2014). Operative agreement on lateral compression-1 pelvis fractures. a survey of 111 OTA members[J]. J Orthop Trauma.

[7] Sagi HC, Coniglione FM, Stanford JH (2011). Examination under anesthetic for occult pelvic ring instability[J]. J Orthop Trauma.

[8] Tosounidis T, Kanakaris N, Nikolaou V, Tan B, Giannoudis PV (2012). Assessment of lateral compression type 1 pelvic ring injuries by intraoperative manipulation: which fracture pattern is unstable?[J]. Int Orthop.

[9] Zhang BF, Zhang H, Wang PF, Wang H, Lei JL, Fu YH, Cong YX, Huang H, Huo XM, Zhuang Y, Zhang K (2017). The role of ultrasonography in examination of the stability of Tile-B2 pelvic fractures: 7 case reports and a literature review[J]. Medicine (Baltimore).

[10] Bruce B, Reilly M, Sims S (2011). OTA highlight paper predicting future displacement of nonoperatively managed lateral compression sacral fractures: can it be done?[J]. J Orthop Trauma.

[11] Van den Bosch EW, Van der Kleyn R, Van Zwienen MC, Van Vugt AB (2002). Nonunion of unstable fractures of the pelvis[J]. Eur J Trauma.

[12] Gautier E, Rommens PM, Matta JM (1996). Late reconstruction after pelvic ring injuries[J]. Injury.

[13] Council for International Organizations of Medical S (2002). International ethical guidelines for biomedical research involving human subjects[J]. Bull Med Ethics.

[14] Tile M (1996). Acute pelvic fractures: I. causation and classification[J]. J Am Acad Orthop Surg.

[15] Tile M (1988). Pelvic ring fractures: should they be fixed?[J]. J Bone Joint Surg Br Vol.

[16] Matta JM, Saucedo T (1989). Internal fixation of pelvic ring fractures[J]. Clin Orthop Relat Res.

[17] Ponsen KJ, Joosse P, Schigt A, Goslings JC, Luitse JS (2006). Internal fracture fixation using the Stoppa approach in pelvic ring and acetabular fractures: technical aspects and operative results[J]. J Trauma.

[18] Gerbershagen HJ, Dagtekin O, Isenberg J, Martens N, Ozgur E, Krep H, Sabatowski R, Petzke F (2010). Chronic pain and disability after pelvic and acetabular fractures—assessment with the Mainz Pain Staging System[J]. J Trauma.

[19] Shlamovitz GZ, Mower WR, Bergman J, Chuang KR, Crisp J, Hardy D, Sargent M, Shroff SD, Snyder E, Morgan MT (2009). How (un)useful is the pelvic ring stability examination in diagnosing mechanically unstable pelvic fractures in blunt trauma patients?[J]. J Trauma.

[20] Olson SA, Pollak AN (1996). Assessment of pelvic ring stability after injury. Indications for surgical stabilization[J]. Clin Orthop Relat Res.

[21] Young JW, Burgess AR, Brumback RJ, Poka A (1986). Pelvic fractures: value of plain radiography in early assessment and management[J]. Radiology.

